# Greatly enhanced hole collection of MoO_*x*_ with top sub-10 nm thick silver films for gridless and flexible crystalline silicon heterojunction solar cells†

**DOI:** 10.1039/d2ra01512a

**Published:** 2022-08-03

**Authors:** Qiyun Lei, Xinan Xu, Na Lu, Liu Yang, Sailing He

**Affiliations:** Centre for Optical and Electromagnetic Research, National Engineering Research Center for Optical Instruments, College of Optical Science and Engineering, Zhejiang University Hangzhou 310058 China optyang@zju.edu.cn sailing@zju.edu.cn; Ningbo Research Institute, Zhejiang University Ningbo 315100 China; JORCEP, School of Electrical Engineering, Royal Institute of Technology (KTH) S-100 44 Stockholm Sweden

## Abstract

Greatly enhanced hole collection of MoO_*x*_ is demonstrated experimentally with a top sub-10 nm thick Ag film, allowing for an efficient dopant-free contacted crystalline silicon (c-Si) heterojunction solar cell without a front grid electrode. With the removal of shadows induced by the front grid electrode, the gridless solar cell with the MoO_*x*_/Ag hole-selective contact (HSC) shows an increment of ∼8% in its power conversion efficiency (PCE) due to the greatly improved short-circuit current density (*J*_sc_) as well as the almost undiminished fill factor (FF) and open-circuit voltage (*V*_oc_), while the gridless solar cells with the conventional MoO_*x*_/ITO and pure MoO_*x*_ HSCs exhibit ∼20% and ∼43% degradations in PCE due to the overwhelming decrease in their FF and *J*_sc_, respectively. Through systematic characterizations and analyses, it is found that the ultrathin Ag film (more conductive than ITO) provides an additional channel for photogenerated holes to transport on MoO_*x*_, contributing to the great enhancement in the hole collection and the great suppression of the shunt loss in the gridless solar cells. A 50 μm thick gridless c-Si heterojunction solar cell with the MoO_*x*_/Ag HSC is 75% thinner but is 86% efficient compared to its 200 μm thick counterpart (while the 50 μm thick gridless solar cell with the MoO_*x*_/ITO HSC is much less efficient). It is over 82% efficient after being bent to a curvature radius as small as 4 mm, also showing superior mechanical flexibility to its counterpart with the MoO_*x*_/ITO HSC. Our MoO_*x*_/Ag double-layer HSC can be easily fabricated through thermal evaporation without breaking the vacuum, saving both the time and cost of the fabrication of the whole device. Therefore, this work provides a guide for the design of efficient HSCs for high-efficiency, low-cost, and flexible solar cells.

## Abstract 2

Greatly enhanced hole collection of MoO_*x*_ is demonstrated experimentally with a top sub-10 nm thick Ag film. With this hole-selective contact, a 50 μm thick gridless c-Si solar cell, showing excellent flexibility, is 75% thinner but 86% efficient.

## Graphical abstract

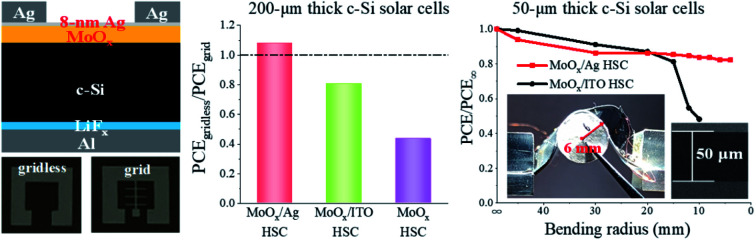

## Introduction

Crystalline silicon (c-Si) solar cells have dominated the worldwide photovoltaic (PV) market for decades. To reduce the levelized cost of electricity, c-Si PV devices are required to constantly increase their power conversion efficiency (PCE) and simultaneously decrease their cost. Recently, remarkable progress has been made owing to advanced passivating contacts.^1–3^ Based on polycrystalline silicon (poly-Si)/tunnel oxide and hydrogenated amorphous silicon (a-Si:H) passivating contacts, the PCE has reached and even surpassed 26%.^4–6^ In these contacts, poly-Si and a-Si:H need to be either p-type or n-type doped to selectively collect holes or electrons. However, doping has its own drawbacks. Physically, both the short-circuit current density (*J*_sc_) and open-circuit voltage (*V*_oc_) will be negatively affected through dopant-induced Auger recombination, bandgap narrowing, surface/interface recombination, and free-carrier absorption.^7–11^ Additionally, both poly-Si and a-Si:H are grown with inflammable and explosive precursor gases. For poly-Si, high-temperature post-annealing (*e.g.*, 900 °C)^4^ is required, which is bound to increase the fabrication cost.

To overcome the above drawbacks, dopant-free carrier-selective contacts have been developed and have quickly attracted much attention.^1–3,11,12^ Electron-selective contacts (ESCs) usually adopt low-workfunction materials (*e.g.*, Ca,^13^ Mg^14^). Alternatively, sub-stoichiometric materials can be introduced to the metal/c-Si interface as passivating layers (*e.g.*, TiO_*x*_,^15–17^ MgO_*x*_,^18^ SiO_*x*_ (ref. 19)) or barrier-lowering layers (*e.g.*, LiF_*x*_ (ref. 20–22) and MgF_*x*_ (ref. 23)) to further reduce the interfacial barrier. In contrast, high-workfunction materials are always chosen as hole-selective contacts (HSCs),^1–3,12,24^*e.g.*, sub-stoichiometric transition-metal oxides (TMOs), including MoO_*x*_,^21,22,25–40^ WO_*x*_,^29,30,41^ V_2_O_*x*_ (ref. 29 and 42–45) and CrO_*x*_.^46^ Based on undoped inorganic HSCs and ESCs, the PCEs of c-Si heterojunction solar cells have exceeded 20%.^47,48^ Meanwhile, these dopant-free heterocontacts can be processed easily under low temperatures, and the PV cost can be reduced further.

TMO-based HSCs are often used as front contacts of solar cells.^22,25,26,28–46^ Due to their rather poor conductivity, TMOs have to be capped with transparent conductive oxides (TCOs),^22,25,26,28–30^ such as indium tin oxide (ITO) and hydrogenated indium oxide, to improve their carrier transport and collection efficiency. Otherwise, the fill factor (FF) of the cell becomes quite low even with dense Ag grids directly on top of the TMO.^49^ For those TMO/TCO-based HSCs, a Ag grid electrode is indispensable,^22,25,26,28–46^ inevitably shading part of the sunlight and decreasing the absorption and thus the *J*_sc_. It is also known that indium is scarce and TCOs containing indium have been increasing in price, especially ITO. Additionally, fragile TCOs are unfriendly to emerging flexible solar cells. To replace these TMO/TCO-based HSCs, TMO/metal/TMO three-layer HSCs have been developed and applied to c-Si solar cells recently.^50–52^ With an ultrathin metallic film (10 nm or less in thickness), such HSCs become highly conductive and the FFs of the c-Si solar cells are significantly improved.^36–38^ All these cells are measured using front Ag grid electrodes, whose shadow effects are not discussed. Further investigation is highly necessary to fully demonstrate the excellent hole transport and collection ability of these HSCs.

In this work, a sub-10 nm thick Ag film is employed on top of MoO_*x*_ to form a double-layer HSC for efficient hole extraction from a c-Si heterojunction solar cell with LiF_*x*_/Al as the back ESC. Compared with previous reports,^36–38^ the outer layer of TMO is not added because the MoO_*x*_/Ag double-layer HSC is simple and is already sufficient to demonstrate the excellent hole transport and collection ability of the metal-incorporated HSC. When the front grid electrode is removed, our solar cell with the MoO_*x*_/Ag HSC shows surprisingly improved PCE due to the enhanced *J*_sc_ and almost undiminished FF and *V*_oc_; while in great contrast, solar cells with the conventional MoO_*x*_/ITO HSC and pure MoO_*x*_ HSC exhibit obvious degradations in PCE due to the overwhelming decrease in FF and *J*_sc_, respectively. Systematic characterization of the three HSCs has been conducted and analyzed. Finally, a 50 μm thick gridless c-Si heterojunction solar cell with the MoO_*x*_/Ag double-layer HSC is demonstrated, which is 75% thinner but retains 86% of the PCE of its 200 μm thick counterpart. It also demonstrates much better mechanical flexibility than its counterpart with the MoO_*x*_/ITO HSC.

## Experimental section

### Fabrication of c-Si heterojunction solar cells

N-Type (100) Czochralski double-side polished c-Si wafers (thickness: 200 μm; resistivity: 1–5 Ω cm) were employed to fabricate the c-Si heterojunction solar cells. Thin solar cells were fabricated on thin c-Si wafers, which were thinned from the 200 μm thick wafers *via* 50 wt% KOH etching at 90 °C, as described in detail in our previous work.^49,53^ Before device fabrication, the original or thinned wafers were thoroughly cleaned and immersed in a dilute 5 wt% hydrofluoric (HF) acid aqueous solution for 60 s to remove the surface oxide generated during the cleaning processes. Immediately after being dried in an oven at 90 °C for 10 min, the wafers were transferred to the vacuum chamber of our thermal evaporator (VNANO VZZ-300S). Another thin oxide layer regrew on the surfaces of the c-Si wafer because air was in the oven. However, it was found that such a drying process was of great necessity to improve the interfacial qualities between c-Si and LiF_*x*_/MoO_*x*_ for deposition, and thereby the power conversion performance of the device (Note S1, ESI†). The rear ESC with ∼2.1 nm thick LiF_*x*_ and 200 nm thick Al was sequentially deposited without breaking the vacuum condition. All the three MoO_*x*_/Ag, MoO_*x*_/ITO, and pure MoO_*x*_ HSCs included a ∼23 nm thick MoO_*x*_ layer, which was thermally evaporated directly onto the front surface of the c-Si wafers. For the MoO_*x*_/Ag HSC, an ultrathin Ag film was evaporated immediately after the MoO_*x*_ deposition without breaking the vacuum condition. The pressure was 1.2 × 10^−4^ Pa. Since the Ag films to be deposited were no thicker than 10 nm, the deposition rate could not be too high, or the thickness would be difficult to control. A very low rate was also not preferred because it was unfavorable for the formation of continuous Ag films under 10 nm in thickness.^54^ Therefore, 0.7 nm s^−1^ was chosen for the deposition of our ultrathin Ag films. For the MoO_*x*_/ITO HSC, a 55 nm thick ITO film was sputtered onto the MoO_*x*_-coated c-Si wafer with a magnetron sputter (Kurt J. Lesker PVD75; DC power: 100 W; argon pressure: 3 mTorr; room temperature). Finally, 200 nm thick Ag electrodes were thermally deposited with either a grid or gridless shadow mask to produce grid or gridless solar cells with different HSCs.

### Characterization

For the characterization of the c-Si solar cells, current density–voltage (*J*–*V*) curves were recorded with a source meter (Keithley 2450) when the solar cells were illuminated with simulated AM1.5 G sunlight produced by a solar simulator (SAN-EI ELECTRIC AAA). The contact resistivity and sheet resistance of the HSCs on c-Si were measured using the TLM method, as schematically shown in the upper left inset of Fig. 2a and described in detail in Note S2, ESI.† The morphologies of the ultrathin Ag films with different thicknesses and the cross-section of the thin c-Si wafer were inspected using SEM (Carl Zeiss Ultra 55). The transmittance spectra were recorded with an integrating sphere-based spectrometer for the HSCs deposited on glass and normalized to the spectrum of the glass. A four-probe system was employed to measure the sheet resistance of the ultrathin Ag films with different thicknesses. Minority carrier lifetimes were measured with a quasi-steady-state photoconductance decay tester (Sinton WCT-120) and the samples were a piece of bare n-type Si wafer and three pieces of n-type Si wafers, which were covered with pure MoO_*x*_, MoO_*x*_/Ag, or MoO_*x*_/ITO HSCs on only one side of each wafer. It is a contactless method for generating carriers with light illumination and detecting the photocarriers through an induction coil embedded in the instrument under the sample.^55^ The interfaces of c-Si/MoO_*x*_, MoO_*x*_/Ag and MoO_*x*_/ITO were observed with an FEI Tecnai G2 F20 S-TWIN TEM instrument. The TEM samples were fabricated by focused ion beam (FIB) milling (Carl Zeiss Quata 3D FEG). The original and etched wafer surfaces were inspected by scanning probe microscopy (SPM, MultiMode VEECO). For the mechanical flexibility measurements, the thin solar cell was carefully attached to a piece of flexible PET substrate, which was fixed to two sample holders. One was fixed and the other was moving, producing different curvature radii for the device on the PET substrate.

## Results and discussion

### Great carrier collection with few nanometer thick Ag films on MoO_*x*_ for gridless c-Si heterojunction solar cells

**Fig. 1:**
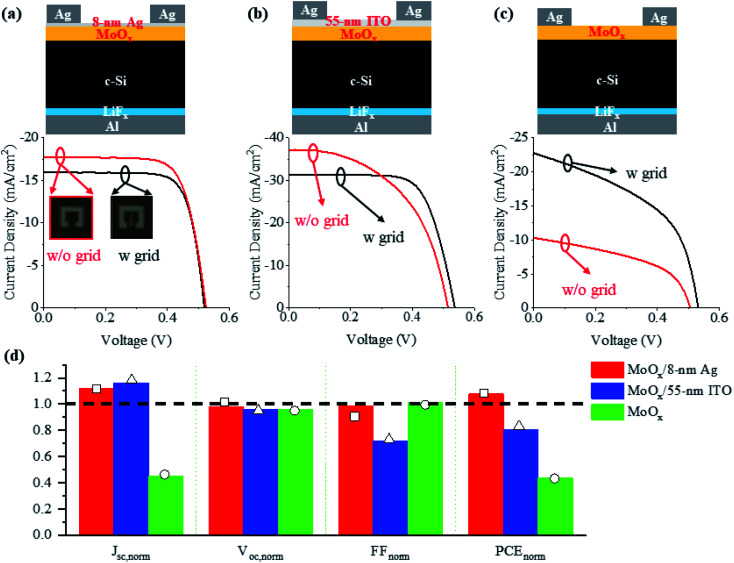
Comparison of c-Si heterojunction solar cells with (a) MoO_*x*_/Ag, (b) MoO_*x*_/ITO, and (c) pure MoO_*x*_ as the HSCs: (top) schematic diagrams and (bottom) the best *J*–*V* curves for the champion cells with (black) and without (red) grid electrodes. (d) Normalized characteristic parameters, *i.e.*, *J*_sc,norm_, *V*_oc,norm_, FF_norm_ and PCE_norm_, calculated from the average values of the three kinds of solar cells without the grid electrodes to those of the counterparts with the grid electrodes. *J*_sc,norm_, *V*_oc,norm_, FF_norm_ and PCE_norm_ calculated from the values corresponding to the champion PCEs of the three kinds of solar cells are indicated by squares, triangles, and circles, respectively.

Fig. 1a shows the schematic diagram of our fabricated c-Si heterojunction solar cell based on dopant-free carrier-selective contacts. The c-Si wafer was 200 μm thick until otherwise specified. The bottom electron-selective contact consists of a ∼2 nm thick LiF_*x*_ film and a ∼100 nm thick Al film, evaporated in sequence without breaking the vacuum. The top hole-selective contact (HSC) consists of a ∼23 nm thick MoO_*x*_ film and a ∼8 nm thick Ag film, also deposited sequentially without breaking the vacuum. The LiF_*x*_, Al and MoO_*x*_ film thicknesses are chosen from our previous work.^53,54^ The ultrathin Ag film is transparent (Fig. S3, ESI†), allowing sunlight to transmit through MoO_*x*_ into the c-Si active layer. Importantly, the sub-10 nm Ag film is able to greatly enhance the hole collection through the bottom MoO_*x*_ film. In order to demonstrate the great hole collection capability of the MoO_*x*_/Ag double-layer HSC, solar cells with top grid and gridless electrodes (with thicknesses of ∼100 nm, shown in the inset of Fig. 1a) were fabricated and compared. For further comparison, the hole collection capabilities of two other conventional HSCs, namely, the MoO_*x*_/ITO double-layer and the pure MoO_*x*_ layer, were also demonstrated with their cell structures schematically shown in Fig. 1b and c, respectively. Here, the MoO_*x*_ films have the same thickness of ∼23 nm, while the ITO layer is ∼55 nm thick for antireflection. For each kind of solar cell, at least three cells were fabricated to evaluate the fabrication repeatability. The light *J*–*V* curves were characterized and the averaged characteristic parameters are summarized in Table 1, where the characteristic parameters of the solar cell with the maximum power conversion efficiency (PCE) are presented below the average values. The light *J*–*V* curves of the champion solar cells with grid and gridless electrodes are plotted below the corresponding schematic diagrams, as shown in Fig. 1a–c, respectively.

**Table 1:** Averaged characteristic parameters of the 200 μm thick grid and gridless c-Si heterojunction solar cells with MoO_*x*_/Ag, MoO_*x*_/ITO, or pure MoO_*x*_ as the HSCs. Each value is calculated from the parameters of more than three cells. For each solar cell, the characteristic parameters of the cell with maximum PCE are presented below the average values

HSCs		*J*_sc_ mA cm^−2^	*V*_oc_ mV	FF%	PCE%	Shunt resistance Ω cm^2^	Series resistance Ω cm^2^
MoO_*x*_/Ag	With grid	15.68 ± 0.29	517 ± 1.7	75.92 ± 0.1	6.16 ± 0.12	1724.3 ± 181.8	2.96 ± 0.14
		15.92	519	75.81	6.27	1828.4	3.01
	Without grid	17.54 ± 0.61	504.7 ± 16.6	75.0 ± 1.42	6.63 ± 0.17	1407.4 ± 294.5	2.84 ± 0.82
		17.69	524.4	72.99	6.77	1243.8	3.99
MoO_*x*_/ITO	With grid	31.35 ± 0.08	531 ± 8.5	68.75 ± 2.24	11.49 ± 0.46	658.1 ± 66.1	2.75 ± 0.09
		31.28	537	70.2	11.8	728.2	2.72
	Without grid	36.46 ± 0.90	510 ± 6.2	49.47 ± 3.05	9.25 ± 0.92	302.1 ± 38.7	2.81 ± 0.32
		37.12	515	51.29	9.81	296.8	2.67
MoO_*x*_	With grid	22.57 ± 0.37	529 ± 5.6	46.67 ± 1.24	5.57 ± 0.17	70.1 ± 2.2	3.69 ± 0.56
		22.68	532	47.75	5.76	68.8	3.18
	Without grid	10.09 ± 0.2	508 ± 7	47.2 ± 0.5	2.42 ± 0.05	141.5 ± 2.3	6.76 ± 0.37
		10.32	506	47.31	2.47	140.4	6.7

From Fig. 1a–c, it can be seen that for the grid solar cells, both the MoO_*x*_/Ag and MoO_*x*_/ITO HSCs enable nearly square *J*–*V* curves with large shunt resistances and small series resistances, indicating high fill factors (FFs), in great contrast to the pure MoO_*x*_ HSC. Especially for the MoO_*x*_/Ag HSC grid cell, due to the good conductivity of the ∼8 nm Ag film, the shunt resistance is maximal, leading to a maximum FF of up to 75.92%. However, due to the absorption of the ultrathin Ag film, the transmittance of the MoO_*x*_/Ag HSC is lower than those of the MoO_*x*_/ITO and the pure MoO_*x*_ HSCs (Fig. S3, ESI†). This results in a much lower short-circuit current density (*J*_sc_) and thus in a degraded open-circuit voltage (*V*_oc_). Therefore, the MoO_*x*_/Ag HSC grid solar cell cannot compete with the MoO_*x*_/ITO HSC grid solar cell (PCE ∼ 11.49%) but still performs better (PCE ∼ 6.16%) than the pure MoO_*x*_ HSC grid solar cell (PCE ∼ 5.57%) because of the rather high FF.

When the inner grid is removed from the top electrode, the three solar cells behave differently (Fig. 1a–c). In order to clearly see the changes, the characteristic parameters of the three gridless solar cells were normalized to those of the grid counterparts and are plotted in Fig. 1d. The normalized parameters for the champion solar cells are also indicated in Fig. 1d. Without the shadow of the grid, the illuminated area is increased by about 9% from 23 to 25 mm^2^. For the MoO_*x*_/Ag and MoO_*x*_/ITO HSC solar cells, the *J*_sc_ values are improved by ∼12% and ∼16%, respectively; while for the pure MoO_*x*_ HSC solar cell, the *J*_sc_ is decreased by ∼55%. This indicates that the double-layer HSCs are much more effective in collecting holes, even those generated in the central illuminated area far from the electrode frame. In contrast, the great reduction in *J*_sc_ for the pure MoO_*x*_ HSC solar cell mainly results from the rather poor conductivity of MoO_*x*_. Despite the increased solar illumination and subsequently increased photocarriers, a large number of holes cannot be efficiently extracted by the electrode frame without the inner grid.

Due to the great reduction in *J*_sc_, the *V*_oc_ is also degraded for the gridless solar cell with the pure MoO_*x*_ HSC. For the solar cells with the MoO_*x*_/Ag and MoO_*x*_/ITO HSCs, because the increase in photocarriers and *J*_sc_ with the absence of the optical shadows is inevitably accompanied by the increase in carrier recombination, the average *V*_oc_ decreases a little (Fig. 1d and Table 1). This can be verified by the smaller minority carrier lifetime for the MoO_*x*_/Ag- and MoO_*x*_/ITO-coated wafers than that for the pure MoO_*x*_-coated wafer (demonstrated in Fig. 3a). Fortunately, the *V*_oc_ degradation is very small for all the solar cells (less than 4%) and does not affect the PCE much. For the gridless MoO_*x*_/Ag HSC solar cell with the champion PCE, its *V*_oc_ is even slightly higher than that of the counterpart with the inner grid electrode (Fig. 1d and Table 1).

For the gridless solar cells with the MoO_*x*_/Ag and pure MoO_*x*_ HSCs, the FFs remain almost unchanged compared with those of the grid cells, but the mechanisms are quite different. In the former, both the shunt and series resistances decrease when the grid is removed. This means that the conductive ultrathin Ag film is favorable for collecting photogenerated holes, but the shunt loss increases without the help of the inner grid for conduction. The opposite behavior happens for the latter because of the less conductive MoO_*x*_ without the grid, leading to increased shunt and series resistances (Table 1). For the gridless solar cell with the MoO_*x*_/ITO HSC, the balance between the hole collection and shunt loss is broken with greatly increased shunt channels. Thus, its FF is greatly reduced (Fig. 1d).

Based on all the above parameters, only the MoO_*x*_/Ag HSC can improve the cell PCE (by ∼8%) when the grid is removed. Great PCE degradations of ∼20% and ∼43% are observed when the conventional MoO_*x*_/ITO and MoO_*x*_ HSCs are applied to the gridless solar cells. Unfortunately, the PCE of the gridless MoO_*x*_/Ag HSC solar cell is still inferior to that of the gridless MoO_*x*_/ITO HSC solar cell due to the much lower *J*_sc_, which is induced by the much lower optical transmissivity of the MoO_*x*_/Ag HSC (Fig. S3, ESI†). That being said, the MoO_*x*_/Ag HSC is more advantageous in fabrication through thermal evaporation without breaking the vacuum, enabling high-throughput and low-cost fabrication of the whole device. However, to fabricate the MoO_*x*_/ITO HSC, pumping twice is necessary for thermal evaporation and sputtering, respectively, which is time-consuming and cost-ineffective. The MoO_*x*_/Ag HSC is also more mechanically flexible than the MoO_*x*_/ITO HSC, allowing for thin and highly flexible c-Si solar cells as emerging wearable power sources (demonstrated below).

### Comprehensive analyses of contact performance

**Fig. 2:**
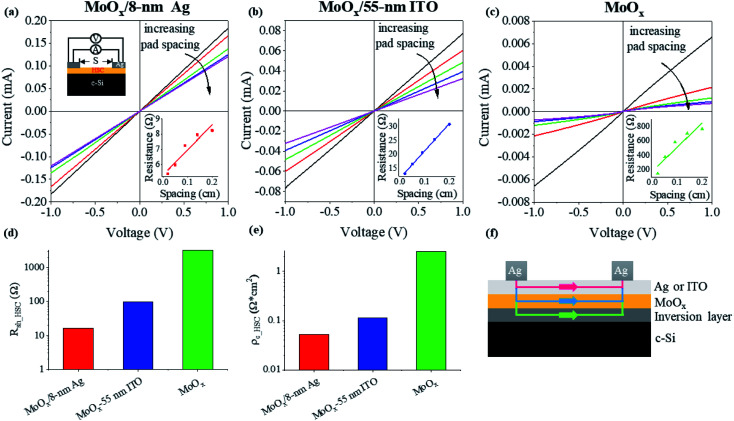
TLM-based HSC/c-Si contact measurement: current–voltage curves between Ag line pad electrodes with different spacings for (a) the MoO_*x*_/Ag, (b) the MoO_*x*_/ITO, and (c) the pure MoO_*x*_ HSCs; (d) extracted sheet resistances, *R*_sh_HSC_, and (e) contact resistivities, *ρ*_c_HSC_, of the three HSCs; (f) schematic diagram of the current flow between two Ag line pad electrodes.

In order to elaborate on the various above photovoltaic behaviors, we characterized the contact performances between the three different HSCs and the n-type c-Si with the transfer length method (TLM).^27^ As schematically shown in the upper left inset of Fig. 2a, a series of current–voltage curves were measured through two Ag line pads on top of the HSCs with various pad spacings, which are plotted in Fig. 2a–c, respectively, for the MoO_*x*_/Ag, MoO_*x*_/ITO, and pure MoO_*x*_ HSCs. All contacts exhibit very good ohmic features. In each panel, the total resistances were also plotted in the lower right inset as a function of the pad spacing and fitted using a straight line, from which the sheet resistance, *R*_sh_HSC_, and the contact resistivity, *ρ*_c_HSC_, of the HSC were calculated and are plotted in Fig. 2d and e, respectively. Details can be found in Note S2, ESI.† Both the *R*_sh_HSC_ and *ρ*_c_HSC_ are drastically reduced when the MoO_*x*_ film is covered with an ultrathin Ag film or an ITO film and the MoO_*x*_/Ag HSC affords the smallest values of *R*_sh_HSC_ and *ρ*_c_HSC_. For all the three HSCs, c-Si is in direct contact with MoO_*x*_, which has a much higher workfunction than c-Si. The Fermi level equilibration makes the conduction and valence bands of c-Si bent up near this contact interface, where electrons are blocked and holes are accumulated to form an inversion layer. Such band alignment is beneficial for hole transportation and collection. Therefore, both the measured *R*_sh_HSC_ and *ρ*_c_HSC_ include the resistances of the whole HSC and the inversion layer. In order to clearly show how holes are transported from one Ag line pad to another, we present a schematic illustration in Fig. 2f, where there are three channels for hole transport at the c-Si/MoO_*x*_/Ag or c-Si/MoO_*x*_/ITO contacts. When current is injected from the left Ag line pad, part of the holes first go vertically through the double-layers of the HSC and then flow horizontally in the inversion layer to the right Ag line pad; part of them go through the top Ag or the ITO layer and into the MoO_*x*_ layer for horizontal transportation; and most holes will flow directly in the top Ag or the ITO layer because of their much better conductivity than MoO_*x*_ or the inversion layer. Due to the absence of the last highly conductive channel in the pure c-Si/MoO_*x*_ contact, both vertical and horizontal transportation will meet extremely high resistances. Therefore, for the pure MoO_*x*_ HSC solar cell, the FF is rather low and a grid is required to collect photogenerated holes, which are otherwise likely to be lost by recombination. Since Ag is more conductive than ITO (to be compared in the next section), the photogenerated holes, which can be collected with lower resistance, can also transport a longer distance. Thus, even for the gridless MoO_*x*_/Ag HSC solar cell, the FF still remains very high and the holes are collected efficiently (Fig. 1c and Table 1).

**Fig. 3:**
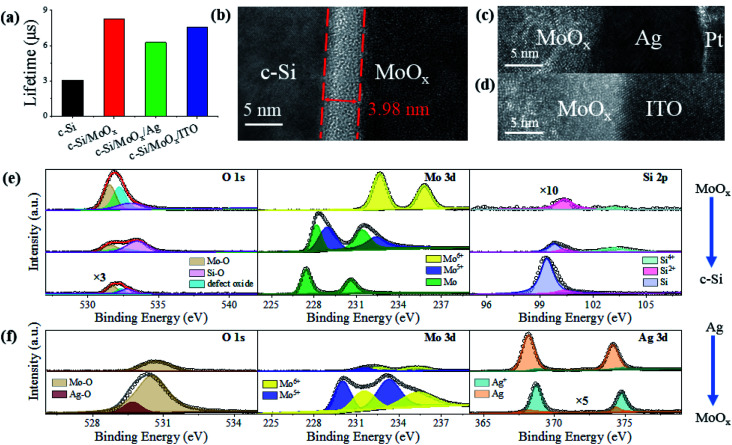
(a) Minority carrier lifetimes of the n-type c-Si, c-Si/MoO_*x*_, c-Si/MoO_*x*_/Ag, and c-Si/MoO_*x*_/ITO samples at an injection level of 10^15^ cm^−3^. TEM images of the (b) c-Si/MoO_*x*_, (c) MoO_*x*_/Ag and (d) MoO_*x*_/ITO interfaces. XPS depth profiling results of (e) the c-Si/MoO_*x*_ interface, showing O 1s, Mo 3d, and Si 2p regions; (f) the MoO_*x*_/Ag interface, showing O 1s, Mo 3d, and Si 2p regions (circles: measured; lines: fitted). Some spectra with extremely small intensities are magnified by different multipliers (indicated close to the curves) so as to be distinguished.

Fig. 3a shows that the measured minority carrier lifetimes of the c-Si wafers covered with the above three HSCs are all higher than that of the individual c-Si wafer. This suggests the passivation effects of the MoO_*x*_ layer, which is however weakened a little bit by the additional Ag or the ITO layer. As mentioned above, due to the large difference in workfunction, an inversion layer forms near the c-Si/MoO_*x*_ interface, where holes are accumulated and electrons are blocked. Consequently, carrier recombination here is reduced, which is known as the field-effect passivation of MoO_*x*_.^49,53^ From the field-emission transmission electron microscopy (TEM) image in Fig. 3b, an interlayer can be clearly observed between MoO_*x*_ and the n-type c-Si regardless of whether MoO_*x*_ is covered with Ag or ITO. The interlayer thickness is ∼3.98 nm. As reported in our previous work,^49,53^ this interlayer, containing Mo, O, and Si elements, is mainly due to the oxidization of the c-Si surface and the reduction of the MoO_*x*_ film. To confirm this point, in this work we explored the interfacial chemical state of the c-Si/MoO_*x*_ interface through depth profiling X-ray photoelectron spectroscopy (XPS). As shown in Fig. 3e, the O 1s spectrum can be deconvoluted into three dominant peaks associated with Mo–O bonds (531.6 eV), Si–O bonds (532.8 eV), and defective oxide (532.2 eV; inherent to many TMOs^56^) on the MoO_*x*_ side. As the etching depth increases, the dominant Mo–O peak drops, while the Si–O peak first increases and then decreases, indicating the oxidization of the Si surface during MoO_*x*_ deposition. The existence of the oxide interlayer at the MoO_*x*_/c-Si interface is also confirmed by the Mo 3d and Si 2p spectra. The Mo 3d spectrum shifts to a lower binding energy, indicating the reduction of Mo^6+^ cations on the MoO_*x*_ side to Mo atoms on the c-Si side, while Si is partially oxidized and a mixture of sub-stoichiometric species coexist in the interlayer. It is fully illustrated that the oxide interlayer is caused by the evaporated MoO_*x*_, which oxidizes the bottom c-Si surface, making it reduced. The oxide interlayer is able to chemically passivate the dangling bonds of the c-Si surface, further improving the minority carrier lifetime (Fig. 3a).

In contrast, no apparent interlayers appear at the MoO_*x*_/Ag and MoO_*x*_/ITO interfaces shown in Fig. 3c and d, respectively. The thermally evaporated ∼8 nm ultrathin Ag film does not look very uniform (whose morphology is demonstrated below) and the interface seems not very flat (Fig. 3c); while the interface between MoO_*x*_ and the sputtered ITO appears flat, and there is no apparent damage during sputtering (Fig. 3d). In order to further explore the interfacial variation at the MoO_*x*_/Ag interface, depth profiling XPS was performed and is illustrated in Fig. 3f. On the Ag side, Ag–O bonds can hardly be distinguished from the O 1s spectrum. However, as the etching depth goes to the MoO_*x*_ side, the Ag–O peak (529.7 eV) appears clearly, meaning that Ag is likely to be oxidized. From the Mo 3d spectra, it is seen that the main peaks move towards the low binding energy, indicating that part of the Mo^6+^ ions are reduced to Mo^5+^ with a significant increase in the intensity on the MoO_*x*_ side. For the Ag 3d spectra, the dominant peaks are first related to Ag atoms in the outer layer and then become associated with Ag^+^ cations in the inner layer. These spectral variations illustrate that the initially evaporated Ag is oxidized upon being deposited onto the MoO_*x*_ film. The remaining Ag atoms at the interface act as recombination centers, leading to a relatively lower carrier lifetime for the c-Si/MoO_*x*_/Ag sample compared to that for the c-Si/MoO_*x*_/ITO sample, as shown in Fig. 3a. Fortunately, such a lifetime decrease does not cause too much degradation in the *V*_oc_ for the photovoltaic devices, as shown in Fig. 1 and Table 1.

### Effects of Ag thickness on carrier collection in the MoO_*x*_/Ag HSC

As a consequence of physical vapor deposition technology, the thermal evaporation of metal films inevitably suffers from the three-dimensional (3D) Volmer–Weber growth mode in the initial stage of deposition.^57–61^ A continuous film with low resistance cannot be achieved with thickness below 10 nm. A thick film will however lead to rather low optical transparency. Fortunately, MoO_*x*_ in this work can serve as a seed layer to mitigate the surface energy difference between the Ag to be deposited and the c-Si active layer. The 3D Volmer–Weber growth mode can thus be suppressed effectively, allowing a sub-10 nm Ag continuous film to be achieved with both low sheet resistance and high transmittance.

**Fig. 4:**
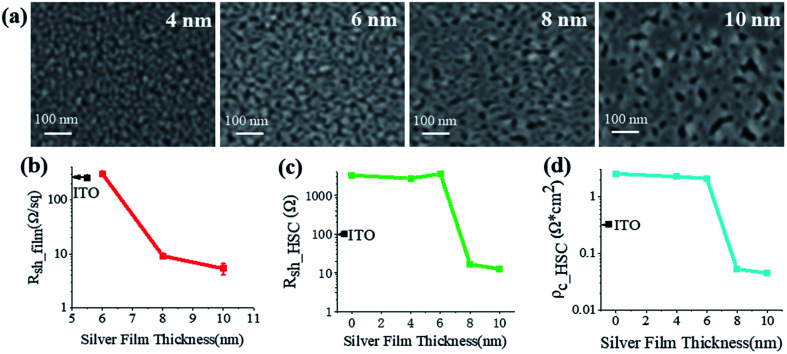
(a) Top view SEM images of our thermally evaporated Ag films with thicknesses of 4, 6, 8, and 10 nm on c-Si/MoO_*x*_ substrates. (b) Thickness-dependent sheet resistance, *R*_sh_film_, of the identical Ag films deposited on quartz/MoO_*x*_ substrates. For each value, at least three samples were fabricated and characterized to show the repeatability of the fabrication. (c) Sheet resistance, *R*_sh_HSC_, and (d) contact resistivity, *ρ*_c_HSC_, of the MoO_*x*_/Ag HSC on n-type c-Si as a function of Ag film thickness. For comparison, the corresponding values of *R*_sh_film_, *R*_sh_HSC_, and *ρ*_c_HSC_ for the ∼55 nm thick ITO are also indicated in (b), (c), and (d), respectively.

Fig. 4a shows the morphological evolution of our thermally evaporated Ag films with different thicknesses on top of the MoO_*x*_-coated c-Si substrates, inspected with scanning electron microscopy (SEM). The thickness-dependent sheet resistances, *R*_sh_film_, and optical transmittance spectra of identical Ag films were characterized on MoO_*x*_-coated quartz substrates and are shown in Fig. 4b and S3, ESI,† respectively. All the MoO_*x*_ coatings were ∼23 nm thick, the same as that applied in solar cells. As shown in Fig. 4a, dense Ag nanoparticles are distributed on the 4 nm thick film. They are separated from each other. Therefore, the Ag film is non-conductive and its *R*_sh_film_ is too large to measure. When the film thickness increases to 6 nm, the small Ag nanoparticles become largely isolated nanoclusters. The *R*_sh_film_ is measurable but is quite large (Fig. 4b). Upon light illumination, localized surface plasmons (LSPs) are excited in either nanoparticles or nanoclusters,^57–61^ resulting in enhanced absorption and thus transmission dips around 650 nm (Fig. S3, ESI†). Further increasing the thickness to 8 nm and 10 nm makes the Ag nanoclusters coalesce into sparse and dense networks, respectively. Due to the increased electron transport paths, the *R*_sh_film_ drops dramatically to below 10 Ω sq^−1^, much smaller than that of the ∼55 nm thick ITO (Fig. 4b). Meanwhile, the LPR-induced transmission dips disappear with improved optical transmittance (Fig. S3, ESI†). Among all the ultrathin Ag films, the 8 nm film shows the best transparency (especially in the near-infrared wavelength range) with a sunlight weighted average transmittance of 55.2% but is much lower than those of the ITO or the uncoated pure MoO_*x*_ films. Fortunately, its rather low *R*_sh_film_ enables extremely good carrier collection performance in terms of the sheet resistance and contact resistivity of the HSC on c-Si, to be described below. The small error bars in the *R*_sh_film_ at various film thicknesses show the very good repeatability of the deposition process.

As shown in Fig. 4c and d, both the sheet resistance, *R*_sh_HSC_, and contact resistivity, *ρ*_c_HSC_, of the MoO_*x*_/Ag HSC follow the trend of the *R*_sh_film_ (rather than the optical transparency), dependent on the Ag film thickness (Fig. 4b). Both the *R*_sh_HSC_ and *ρ*_c_HSC_ drop dramatically when the Ag film thickness changes from 6 to 8 nm. This strong correlation indicates the dominant role of the ultrathin Ag film on the whole HSC regarding carrier collection. Compared with the pure MoO_*x*_ HSC, the top Ag film provides another channel to conduct holes. If it is continuous, *e.g.*, in the 8 and 10 nm thick films, the carrier collection can naturally be improved both vertically and horizontally; while for the discontinuous Ag films with thicknesses below 8 nm, both the *R*_sh_HSC_ and *ρ*_c_HSC_ show no obvious improvement compared with the pure MoO_*x*_ HSC (Fig. 4c and d). Due to the large *R*_sh_film_ of ITO (Fig. 4b), the MoO_*x*_/ITO HSC shows inferior carrier collection ability in terms of its *R*_sh_HSC_ (Fig. 4c) and *ρ*_c_HSC_ (Fig. 4d) compared to the MoO_*x*_/Ag HSCs with Ag film thicknesses of 8 and 10 nm, whose Ag films are more conductive. It is also found that our MoO_*x*_/8 nm Ag HSC owing to a quite low *ρ*_c_HSC_ outperforms almost all the HSCs reported previously (Table S2, ESI†). Therefore, it is believed to be a quite good dopant-free contact for hole collection.

### 50 μm thick gridless and flexible c-Si solar cell with the MoO_*x*_/Ag HSC

**Fig. 5:**
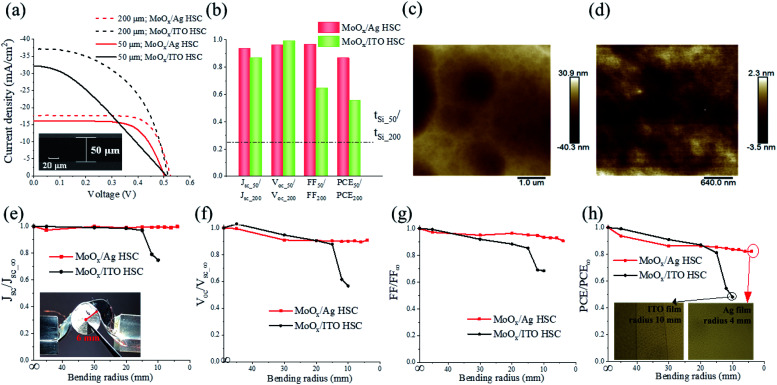
(a) Measured *J*–*V* curves of the 50 μm thick gridless c-Si heterojunction solar cells with the MoO_*x*_/8 nm Ag and MoO_*x*_/55 nm ITO HSCs in comparison with their 200 μm thick counterparts. Inset: SEM image of the 50 μm thick c-Si wafer. (b) Ratios of the characteristic parameters of the 50 μm thick gridless solar cells to those of their 200 μm thick counterparts, *i.e.*, *J*_sc_50_/*J*_sc_200_, *V*_oc_50_/*V*_oc_200_, FF_50_/FF_200_, and PCE_50_/PCE_200_. The wafer thickness ratio, *t*_Si_50_/*t*_Si_200_, is also indicated as a dashed line. Surface morphologies of (c) the etched 50 μm thick and (d) the original 200 μm thick c-Si wafers. Normalized characteristic parameters of the bent gridless solar cells with the MoO_*x*_/Ag and MoO_*x*_/ITO HSCs to those of the unbent counterparts, *i.e.*, (e) *J*_sc_/*J*_sc∞_, (f) *V*_oc_/*V*_oc∞_, (g) FF/FF_∞_, and (h) PCE/PCE_∞_, as a function of bending radius. The inset in (e) shows a gridless solar cell with the MoO_*x*_/Ag HSC bent at a radius of 6 mm; the inset in (h) shows the optical pictures of the 55 nm thick ITO surface after being bent at a radius of 10 mm and of the 8 nm thick Ag film surface after being bent at a radius of 4 mm.

In order to further demonstrate the advantage of the MoO_*x*_/8 nm Ag HSC, we fabricated flexible c-Si heterojunction solar cells based on 50 μm thick wafers (inset of Fig. 5a), which were etched from 200 μm thick as-bought wafers (Experimental section). For comparison, a 50 μm thick flexible solar cell with the MoO_*x*_/55 nm ITO HSC was also fabricated. The *J*–*V* curves of the two flexible solar cells are presented in Fig. 5a and compared with those of the 200 μm thick counterparts. Their characteristic parameters were extracted and are listed in Table 2. In order to clearly show the influence of the wafer thickness, the ratios of the characteristic parameters of the 50 μm thick cell to those of the 200 μm thick cell, *i.e.*, *J*_sc_50_/*J*_sc_200_, *V*_oc_50_/*V*_oc_200_, FF_50_/FF_200_, and PCE_50_/PCE_200_, were calculated and are plotted in Fig. 5b, where the c-Si thickness ratio, *t*_Si_50_/*t*_Si_200_, is also indicated for comparison. When the c-Si thickness, *t*_Si_, is reduced by 75% from 200 μm to 50 μm, for the MoO_*x*_/Ag HSC solar cell, the PCE still retains 86% of the initial value of the 200 μm thick cell with degradation of merely 14%, resulting from the 7%, 4%, and 4% decreases in *J*_sc_, *V*_oc_, and FF, respectively (Fig. 5b). However, due to the larger reduction in *J*_sc_ (13%) and FF (36%), the PCE decreases more dramatically by 45% for the MoO_*x*_/ITO HSC solar cell. Its absolute PCE (5.43%) was even slightly lower than that of the thin MoO_*x*_/Ag HSC solar cell (5.85%).

**Table 2:** Characteristic parameters of the 50 and 200 μm thick c-Si heterojunction solar cells with the MoO_*x*_/8 nm Ag and MoO_*x*_/55 nm ITO HSCs

HSCs	*t*_Si_ (μm)	*J*_sc_ (mA cm^−2^)	*V*_oc_ (mV)	FF (%)	PCE (%)	Shunt resistance (Ω cm^2^)	Series resistance (Ω cm^2^)
MoO_*x*_/Ag	200	17.69	524.4	72.99	6.77	1243.8	3.99
	50	16.51	503	70.43	5.85	925.93	4.79
MoO_*x*_/ITO	200	37.12	515	51.29	9.81	298	2.67
	50	32.19	510	33.08	5.43	154.32	11.85

When the c-Si is thinned, there must be some photons not fully absorbed, especially those in the near-infrared wavelength range. Due to the highly reflective LiF_*x*_/Al ESC, they will be reflected towards the front HSC. Since the MoO_*x*_/ITO HSC is more transparent than the MoO_*x*_/Ag HSC, more light will be transmitted through the MoO_*x*_/ITO HSC into the air, leading to larger reflectivity. In contrast, the MoO_*x*_/Ag HSC benefits the light trapping^62^ and thus the reduced reflectivity of the thin cell (Fig. S3 and S4, ESI†). Therefore, the *J*_sc_ degradation for the MoO_*x*_/Ag HSC solar cell is smaller than that for the MoO_*x*_/ITO HSC solar cell (Fig. 5b).

Because of the very fast etching process, the c-Si surface roughness increased from 0.75 nm to 8.02 nm (Fig. 5c and d), which must lead to enhanced carrier recombination at the c-Si surfaces. Fortunately, owing to the good passivation effects of the HSC and ESC, as demonstrated in Fig. 3, in this work and in our previous work,^49^ the *V*_oc_ values of both solar cells with the MoO_*x*_/Ag and MoO_*x*_/ITO HSCs are not affected much by the rough surface. The relatively larger *V*_oc_ degradation for the MoO_*x*_/Ag HSC solar cell is likely due to the Ag atoms occurring at the MoO_*x*_/Ag interface as recombination centers, as discussed in Fig. 3. In contrast, the rough c-Si surface seems to affect the quality of the sputtered ITO significantly, leading to a significant increment in the series resistance and an apparent decrease in the shunt resistance of the device (Table 2). Therefore, FF for the MoO_*x*_/ITO HSC solar cell drops by 36%, much larger than the FF degradation of 4% for the MoO_*x*_/Ag HSC solar cell (Fig. 5b). This indicates the more excellent tolerance of the ultrathin Ag film to the substrate surface to be deposited.

Furthermore, a mechanical flexibility test was conducted for the two kinds of 50 μm thick solar cells with the setup shown in the inset of Fig. 5e. The measured *J*–*V* curves of the solar cells under different curvature radii are plotted in Fig. S5, ESI.† To show the performance variation with the curvature radius, the characteristic parameters of the bent solar cells were normalized to those of the unbent cells and are plotted in Fig. 5e–h. It can be seen that all the characteristic parameters for the MoO_*x*_/Ag HSC solar cell are above 82% of those for the unbent counterpart, even when the bending radius is reduced to as small as 4 mm. There are no apparent cracks at the Ag surface (inset of Fig. 5g). In great contrast, all the normalized parameters for the MoO_*x*_/ITO HSC solar cell fail to remain higher than 80% but instead drop dramatically when the bending radius becomes smaller than 15 mm. We observed obvious cracks at the 55 nm thick ITO surface at a curvature radius of 10 mm (inset of Fig. 5g), which were the main reason for the failure of the MoO_*x*_/ITO HSC solar cell at small bending radii. Such a comparison fully illustrates the superior mechanical flexibility of the 8 nm thick Ag film, enabling flexible c-Si solar cells as emerging wearable power sources.

## Conclusions

In summary, greatly enhanced hole collection of MoO_*x*_ has been demonstrated experimentally with a top sub-10 nm thick Ag film, which enables an efficient undoped contacted c-Si heterojunction solar cell without a front grid electrode. Compared with solar cells with front grid electrodes, the gridless solar cell with the MoO_*x*_/Ag HSC shows an increment of ∼8% in PCE due to the shadow removal-induced improvement of the *J*_sc_ as well as the almost-undiminished FF and *V*_oc_, while in great contrast, the gridless solar cells with the conventional MoO_*x*_/ITO HSC and pure MoO_*x*_ HSC exhibit obvious degradations in PCE (∼20% and ∼43%, respectively) due to the overwhelming decrease in their FF and *J*_sc_, respectively. Systematic characterizations of the three HSCs have been conducted. It is found that the more conductive ultrathin Ag film (rather than ITO) provides an additional channel for photogenerated holes to transport more quickly on MoO_*x*_, which contributes to the great enhancement in hole collection and shunt loss suppression in the gridless solar cells. Our MoO_*x*_/Ag double-layer HSC can be easily fabricated through thermal evaporation without breaking the vacuum, saving both the time and cost of the fabrication of the whole device. Based on this HSC, a 50 μm thick gridless c-Si heterojunction solar cell is demonstrated, which is 75% thinner but retains 86% of the PCE of its 200 μm thick counterpart (while the 50 μm thick gridless solar cell with the MoO_*x*_/ITO HSC is much less efficient). It is over 82% efficient after being bent to a curvature radius as small as 4 mm, showing much better mechanical flexibility than its counterpart with the MoO_*x*_/ITO HSC. This work provides a guide for the design of high-efficiency and low-cost solar cells, promising for applications as emerging wearable power sources.

## Conflicts of interest

There are no conflicts to declare.

## Supplementary Material

RA-012-D2RA01512A-s001
