# Supplementary material for: Greatly enhanced hole collection of MoOx with top sub-10 nm thick silver films for gridless and flexible crystalline silicon heterojunction solar cells
Source: RSC Adv. 2022 Aug 3;12(33):21482–92. doi: 10.1039/d2ra01512a (PMC9346987; doi:10.1039/d2ra01512a)
Supplement: RA-012-D2RA01512A-s001 [file RA-012-D2RA01512A-s001.pdf]

Electronic Supplementary Material (ESI) for RSC Advances.
This journal is © The Royal Society of Chemistry 2022

Supporting Information

Greatly enhanced hole collection of MoOx with top sub-10 nm thick silver films for gridless and flexible crystalline silicon heterojunction solar cells

Qiyun Lei,[a, †] Xinan Xu,[a, †] Na Lu,[a] Liu Yang*[a,b] and Sailing He*[a,b,c]

[a.] Centre for Optical and Electromagnetic Research, National Engineering Research Center for Optical Instruments, Zhejiang University, Hangzhou 310058, China. E-mails: optyang@zju.edu.cn; sailing@zju.edu.cn

[b.] Ningbo Research Institute, Zhejiang University, Ningbo 315100, China.

[c.] JORCEP, School of Electrical Engineering, Royal Institute of Technology (KTH), S-100 44 Stockholm, Sweden.

Note S1. Demonstration of the importance of the drying process in an air-oven at 90 ℃ for 10 min after HF etching

After thorough cleaning, an oxide layer was produced on the surfaces of the c-Si wafer and removed by the following HF etching. Subsequently, before being transferred to the vacuum chamber of the thermal evaporator, the wafer was dried in an oven at 90 ℃ for 10 min. Since air was in the oven, another oxide thin layer must regrow on the surfaces of the wafer. In order to verify the necessity of this drying process, we fabricated two c-Si heterojunction solar cells with and without the drying process. The HSCs of the two cells were MoO_x/8-nm Ag HSCs and the ESCs were LiF_x/Al ESCs. The measured J-V curves are shown in Fig. S1 and the characteristic parameters are listed in Table S1. With the drying process, the fabricated solar cell demonstrates enhancements in J_{sc}, V_{oc} and PCE with a negligible reduction in FF. This clearly illustrates that the drying process is of great necessity to improve the interfacial qualities between c-Si and LiF_x as well as between c-Si and MoO_x, and hence the power conversion performance of the device.

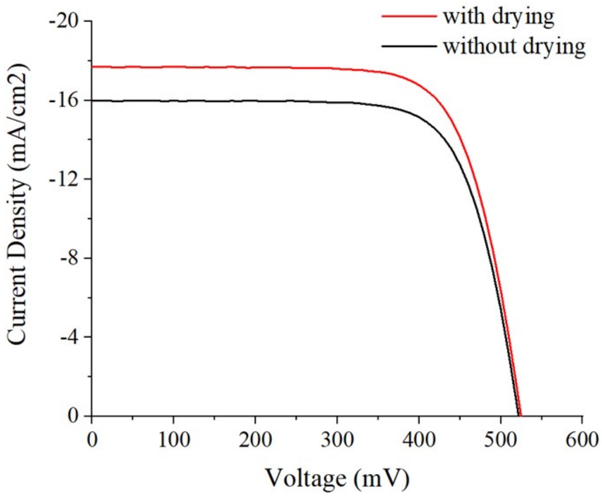

Fig. S1 Measured J-V curves of the 200-μm thick c-Si heterojunction solar cells fabricated with and without the drying process.

Table S1 The characteristic parameters of the 200-μm thick c-Si heterojunction solar cells fabricated with and without the drying process extracted from the measured J-V curves shown in Fig. S1.

200-μm thick c-Si solar cells	J_{sc} (mA/cm²)	V_{oc} (mV)	FF (%)	PCE (%)	Shunt Resistance (Ω·cm²)	Series Resistance (Ω·cm²)
With drying	17.69	524.4	72.99	6.77	1243.8	3.99
Without drying	16	522.1	73.2	6.11	1154.7	3.88

Note S2. Transfer length method

The contact property between the hole-selective contact (HSC) and the n-type Si was characterized with a transfer length method [S1]. As shown in Fig. S2, when a voltage (V) is applied to the Ag line pads, a current (I) can be measured, flowing from one pad to another. If Ohmic contacts are formed at the HSC/Si interfaces, the current will vary linearly with the voltage. The total resistance, R_{tot}, between the two pads can be derived easily from the slope of the linear I-V curve.

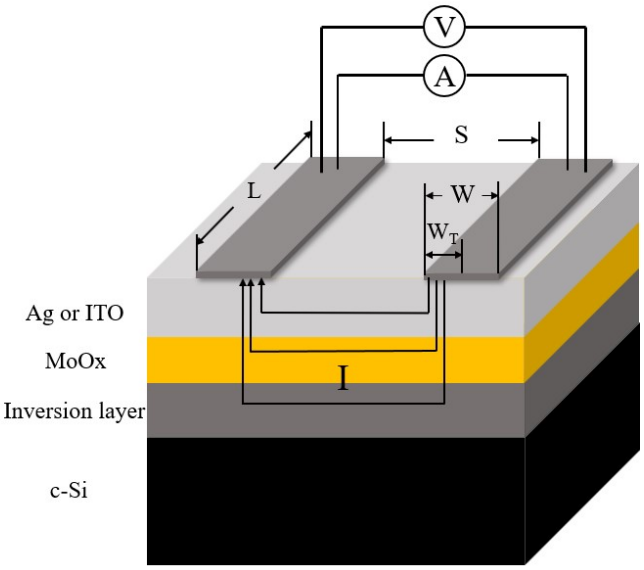

Fig. S2. Schematic diagram of the transfer length method for contact property characterization.

Actually, R_{tot} originates mainly from the in-plane resistance, R_{ip}, between the pads and the contact resistance, R_c, under the pads. Assuming the same R_c of the two pads, R_{tot} is expressed below:

$$R_{tot} = \frac{V}{I} = R_{ip} + 2R_c \tag{1}$$

where, $R_{ip} = R_{sh\_HSC} \bullet S/L$, R_{sh_HSC} is the sheet resistance between the two pads, S and L are the pad spacing and length, respectively. Since the current can pass through the single layer or double layers of the HSC and also the inversion layer immediately under the HSC, R_{sh_HSC} is an overall sheet resistance of both the HSC and the inversion layer.

At the HSC/Si interface, if R_c is small enough, only part of the pad is used for current flow. In this case, the effective width, W_T, is smaller than the physical width, W, of the pad (Fig. S2), and R_c can be expressed below:

$$R_c = \frac{\rho_{c\_HSC}}{W_T L} = \frac{\sqrt{\rho_{c\_HSC} R_{sh\_HSC}}}{L} \tag{2}$$

On the other hand, if W_T is larger than W, the whole pad is used for current flow, and R_c is expressed below:

$$R_c = \frac{\rho_{c\_HSC}}{WL} \tag{3}$$

In Eqs. (2) and (3), ρ_{c_HSC} is the contact resistivity of the HSC/Si interface, which is also an overall

parameter representing all the factors along the current flow vertically from the Ag pad to the HSC under the pad and to the inversion layer immediately under the HSC.

By changing the pad spacing, S, we can measure a series of linear I-V curves and plot the extracted R_{tot} as a function of S, as shown in Fig. 2a, 2b and 2c for the three HSCs. According to Eqs. (1)-(3), R_{tot} varies linearly with S, and can be fitted with a straight line. From the slope and the intersection with the vertical axis of the R_{tot}-S fitting line, both R_{sh_HSC} and ρ_{c_HSC} can be easily derived, respectively.

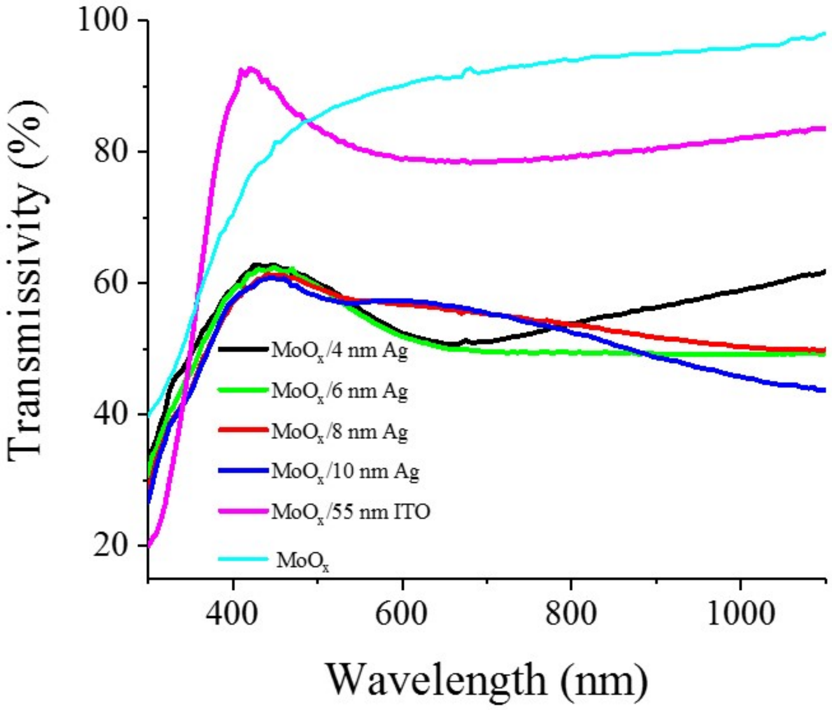

Fig. S3. Measured optical transmission spectra of Ag films with different thicknesses a ~ 55-nm thick ITO on quartz substrates coated with ~ 23-nm thick MoO_x layers. All the spectra are normalized to that of the quartz substrate.

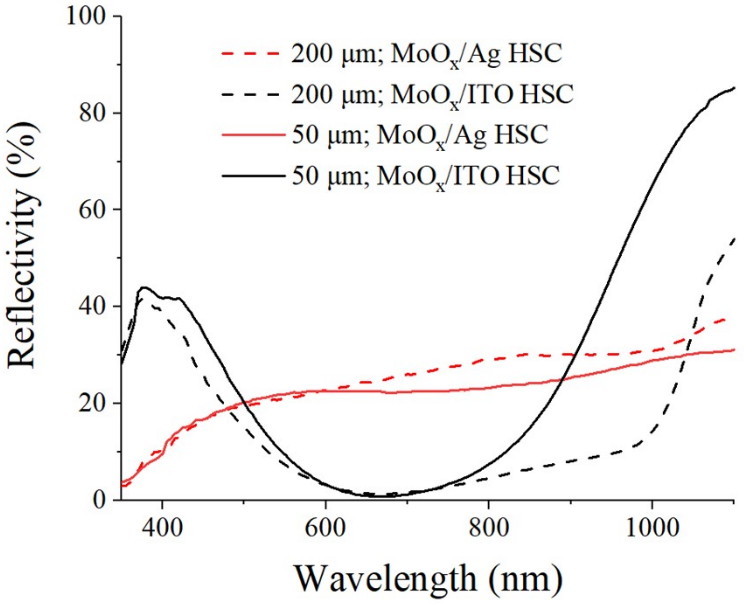

Fig. S4. Measured reflectivity spectra of the 50- and 200-μm thick c-Si heterojunction solar cells with MoO_x/8-nm Ag and MoO_x/55-nm ITO HSCs.

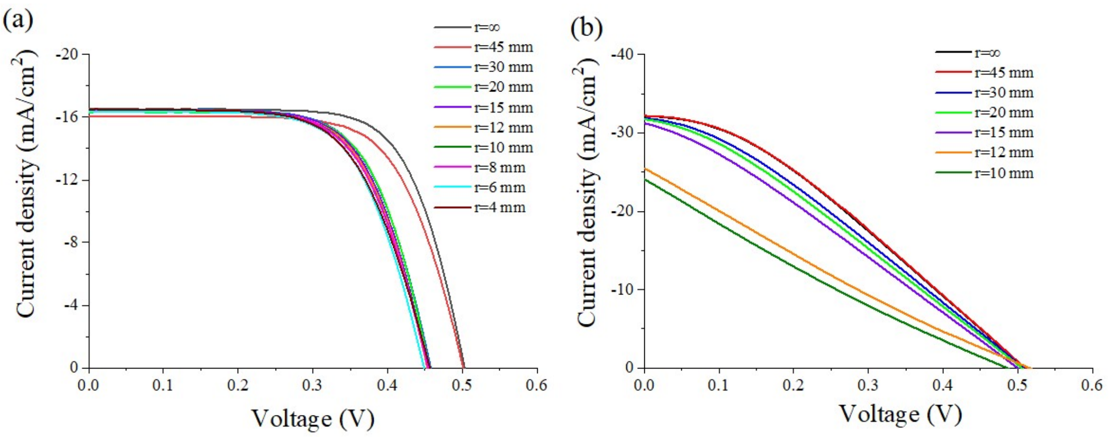

Fig. S5. Measured J-V curves of the 50-μm thick c-Si heterojunction solar cells with (a) the MoO_x/8-nm Ag HSC and (b) the MoO_x/55-nm ITO HSC, being bent at different curvature radii, r.

Table S2 Comparison of the contact resistivities of the MoO_x/8-nm Ag, MoO_x/55-nm ITO, and pure MoO_x HSCs to the n-type c-Si in our work, as well as the contact resistivities of previously-reported HSCs to either n-type or p-type c-Si.

**HSCs**	**ρ_{c_HSC} (Ω*cm^2)**	**References**
8-nm Ag/MoO_x/n-Si	0.053	This work
55-nm ITO/MoO_x/n-Si	0.114	This work
MoO_x/n-Si	2.56	This work
MoO_x/n-Si	0.1	S1
Ag/MoO_{3-x}:V/n-Si	2.1	S2
Ag/ITO/MoO_x/n-Si	0.385	S3
Ag/MoO_x/a-Si:H(i)/n-Si	0.224	S4
MoO_x/n-Si	0.0785	S5
Ag/MoO_x/n-Si	0.0559	S6
Ag/s-MoO_x[a]/n-Si	5.6	S7
Ag/ALD[b] MoO_x/p-Si	0.38	S8
MoO_x/p-Si	0.32	S9
Al/Ni/MoO_x/p-Si	<0.01	S10
MoO_x/p-Si	0.053	S11
ALD[b] VO_x/n-Si	0.095	S12
ALD[b] V_2O_5/n-Si	0.25	S13
Ag/WO_x/V_2O_x/p-Si	0.061	S14
Ag/Cu:CrO_x/p-Si	0.095	S15

[a]Solution-processed MoO_x; [b]Atomic layer deposition.

References

[S1] J. Bullock, A. Cuevas, T. Allen and C. Battaglia, *Applied Physics Letters*, 2014, **105**, 232109.

[S2] C. Liu, L. Zhang, G. Yu, T. Wang, X. Wu, L. Xu, P. Lin, C. Cui, X. Yu and P. Wang, Materials Science in Semiconductor Processing, 2022, 146, 106687.

[S3] M. Nayak and V. K. Komarala, IEEE Transactions on Electron Devices, 2022, 69, 3251-3257.

[S4] W. Wang, J. He, D. Yan, W. Chen, S. Pheng Phang, C. Samundsett, S. Krishna Karuturi, Z. Li, Y. Wan and W. Shen, Solar Energy, 2022, 231, 203-208.

[S5] J. Chen, C. Liu, S. Xu, P. Wang, X. Ge, B. Han, Y. Zhang, M. Wang, X. Wu, L. Xu, P. Lin, X. Huang, X. Yu and C. Cui, Materials Science in Semiconductor Processing, 2021, 132, 105920.

[S6] M. Gülnahar, H. Nasser, A. Salimi and R. Turan, Journal of Materials Science: Materials in Electronics, 2021, 32, 1092-1104.

[S7] C. Lu, Rusli, A. B. Prakoso and Z. Li, Materials Chemistry and Physics, 2019, 236, 121779.

[S8] T. Pan, J. Li, Y. Lin, Z. Xue, Z. Di, M. Yin, J. Wang, L. Lu, L. Yang and D. Li, Journal of Materials Science: Materials in Electronics, 2021, 32, 3475-3486.

[S9] Y. Jiang, S. Cao, L. Lu, G. Du, Y. Lin, J. Wang, L. Yang, W. Zhu and D. Li, Nanoscale Research Letters, 2021, 16, 87.

[S10] G. Gregory, C. Luderer, H. Ali, T. S. Sakthivel, T. Jurca, M. Bivour, S. Seal and K. O. Davis, Advanced Materials Interfaces, 2020, 7, 2000895.

[S11] H. Nasser, F. Es, M. Zolfaghari Borra, E. Semiz, G. Kökbudak, E. Orhan and R. Turan, Progress in Photovoltaics: Research and Applications, 2021, 29, 281-293.

[S12] X. Yang, H. Xu, W. Liu, Q. Bi, L. Xu, J. Kang, M. N. Hedhili, B. Sun, X. Zhang and S. De Wolf, Advanced Electronic Materials, 2020, 6, 2000467.

[S13] G. Masmitjà, E. Ros, R. Almache-Hernández, B. Pusay, I. Martín, C. Voz, E. Saucedo, J. Puigdollers and P. Ortega, Solar Energy Materials and Solar Cells, 2022, 240, 111731.

[S14] Z. Liu, W. Lin, Z. Chen, D. Chen, Y. Chen, H. Shen and Z. Liang, Advanced Materials Interfaces, 2022, 9, 2102374.

[S15] Z. Xu, S. Peng, H. Lin, S. Tian, Z. Wang, J. He, L. Cai, J. Hou and P. Gao, Solar RRL, 2021, 5, 2100064.
